# Causes of Acute Hospitalization in Adolescence: Burden and Spectrum of HIV-Related Morbidity in a Country with an Early-Onset and Severe HIV Epidemic: A Prospective Survey

**DOI:** 10.1371/journal.pmed.1000178

**Published:** 2010-02-02

**Authors:** Rashida A. Ferrand, Tsitsi Bandason, Praise Musvaire, Natasha Larke, Kusum Nathoo, Hilda Mujuru, Chiratidzo E. Ndhlovu, Shungu Munyati, Frances M. Cowan, Diana M. Gibb, Elizabeth L. Corbett

**Affiliations:** 1 London School of Hygiene & Tropical Medicine, London, United Kingdom; 2 Biomedical Research & Training Institute, Harare, Zimbabwe; 3 University of Zimbabwe, Harare, Zimbabwe; 4 University College London, London, United Kingdom; 5 Medical Research Council Clinical Trials Unit, London, United Kingdom; University of the Witwatersrand, South Africa

## Abstract

**Background:**

Survival to older childhood with untreated, vertically acquired HIV infection, which was previously considered extremely unusual, is increasingly well described. However, the overall impact on adolescent health in settings with high HIV seroprevalence has not previously been investigated.

**Methods and Findings:**

Adolescents (aged 10–18 y) systematically recruited from acute admissions to the two public hospitals in Harare, Zimbabwe, answered a questionnaire and underwent standard investigations including HIV testing, with consent. Pre-set case-definitions defined cause of admission and underlying chronic conditions. Participation was 94%. 139 (46%) of 301 participants were HIV-positive (median age of diagnosis 12 y: interquartile range [IQR] 11–14 y), median CD4 count = 151; IQR 57–328 cells/µl), but only four (1.3%) were herpes simplex virus-2 (HSV-2) positive. Age (median 13 y: IQR 11–16 y) and sex (57% male) did not differ by HIV status, but HIV-infected participants were significantly more likely to be stunted (*z*-score<−2: 52% versus 23%, *p*<0.001), have pubertal delay (15% versus 2%, *p*<0.001), and be maternal orphans or have an HIV-infected mother (73% versus 17%, *p*<0.001). 69% of HIV-positive and 19% of HIV-negative admissions were for infections, most commonly tuberculosis and pneumonia. 84 (28%) participants had underlying heart, lung, or other chronic diseases. Case fatality rates were significantly higher for HIV-related admissions (22% versus 7%, *p*<0.001), and significantly associated with advanced HIV, pubertal immaturity, and chronic conditions.

**Conclusion:**

HIV is the commonest cause of adolescent hospitalisation in Harare, mainly due to adult-spectrum opportunistic infections plus a high burden of chronic complications of paediatric HIV/AIDS. Low HSV-2 prevalence and high maternal orphanhood rates provide further evidence of long-term survival following mother-to-child transmission. Better recognition of this growing phenomenon is needed to promote earlier HIV diagnosis and care.

*Please see later in the article for the Editors' Summary*

## Abstract 2

Rashida Ferrand and colleagues show that HIV infection is the commonest cause of hospitalization among adolescents in a high HIV prevalence setting.

## Editors' Summary

**Background:**

Acquired immunodeficiency syndrome (AIDS) has killed more than 25 million people since 1981, and more than 30 million people are now infected with the human immunodeficiency virus (HIV) that causes AIDS. HIV destroys the cells in the immune system that normally provide protection against disease-causing organisms. Consequently, people infected with HIV are susceptible to so-called opportunistic infections, including tuberculosis and pneumonia. HIV is most commonly spread through unprotected sex with an infected partner but another major route of transmission is mother-to-child (vertical transmission) during pregnancy or delivery or during breast feeding. Mother-to-child transmission can be prevented by giving antiviral drugs to HIV-positive mothers during their pregnancy and to their newborn children. But, although most mothers in developed countries have access to this intervention, fewer than half of HIV-positive mothers in low- and middle-income countries receive this treatment and, every year, nearly half a million children become infected with HIV.

**Why Was This Study Done?:**

It is generally thought that HIV infections in infants progress rapidly and that half of the children who acquire HIV from their mothers will die before their second birthday if not treated with antiretroviral drugs. However, as the AIDS epidemic matures, more children are surviving to adolescence with untreated, vertically acquired HIV infection in sub-Saharan Africa, the region where most children with HIV/AIDS live. Little is known about the burden of HIV infection and its contribution to illness and death in adolescents in sub-Saharan Africa but this information is needed to help health care providers prepare for this new aspect of the AIDS epidemic. In this study, the researchers examine the causes of acute hospital admissions (admissions for conditions with a sudden onset and usually a short course) among adolescents in Zimbabwe, a country where the HIV epidemic started early and where one in seven adults is HIV-positive and more than 17,000 children are infected with HIV every year, mainly through vertical transmission.

**What Did the Researchers Do and Find?:**

The researchers recruited 301 10–18-year olds who were admitted to each of the two public hospitals in Harare (Zimbabwe) for acute illnesses between September 2007 and April 2008. Each patient completed a questionnaire about themselves and their health and underwent standard investigations, including HIV testing. Nearly half the participants were HIV positive; about a quarter of these HIV-positive individuals only found out about their status during the study. HIV-positive participants were more likely to be stunted, to have pubertal delay, and to be maternal orphans or have an HIV-infected mother than HIV-negative participants. 69% of HIV-positive participants were admitted to hospital because of infections, often tuberculosis or pneumonia whereas only 19% of the HIV-negative participants were admitted for infections. More than a quarter of all the participants had underlying heart, lung, or other chronic conditions. Finally, 22% of the HIV-positive participants died while in hospital compared to only 7% of the HIV-negative participants. Factors that increased the risk of death among all the participants were advanced HIV infection, pubertal immaturity, and chronic conditions.

**What Do These Findings Mean?:**

These findings indicate that HIV infection is the commonest cause of acute adolescent admission to hospital in Harare (and probably elsewhere in Zimbabwe). Most of these admissions are due to opportunistic infections similar to those seen in HIV-positive adults and to long-term complications of having HIV/AIDS as an infant such as delayed puberty. Other findings indicate that most of the HIV-positive adolescents who participated in this study were infected via vertical transmission, which supports the idea that long-term survival after vertical infection is possible. Because the AIDS epidemic started early in Zimbabwe, there is likely to be a lag before adolescent survivors of vertical HIV transmission become common elsewhere. Nevertheless, all African countries and other places where HIV infection in adults is common need to recognize that the burden of HIV in their acutely unwell adolescents is likely to increase over the next few years. To deal with this emerging aspect of the AIDS epidemic, measures must be introduced to ensure early diagnosis of HIV in this previously neglected age group so that treatment can be started before HIV-positive adolescents become critically ill.

**Additional Information:**

Please access these Web sites via the online version of this summary at http://dx.doi.org/10.1371/journal.pmed.1000178.

- This study is further discussed in a *PLoS Medicine* Perspective by Glenda Gray
- Information is available from the US National Institute of Allergy and Infectious Diseases on HIV infection and AIDS
- HIV InSite has comprehensive information on all aspects of HIV/AIDS, including a list of articles and other sources of information about the primary care of adolescents with HIV
- Information is available from Avert, an international AIDS charity on many aspects of HIV/AIDS, including information on the HIV and AIDS in Zimbabwe, and on children, HIV, and AIDS (in English and Spanish)
- UNICEF also has information about children and HIV and AIDS (in several languages)

## Introduction

In industrialised countries trauma and behavioural disorders, such as substance abuse, obesity, and sexually transmitted infections account for most adolescent morbidity [1]. Chronic diseases, trauma, oncology, and mental disorders account for the majority of hospital admissions [2],[3]. In Africa, research on adolescent morbidity has been limited and has predominantly focused on reproductive health, reflecting the high incidence of sexually transmitted infections and obstetric problems among young people [4],[5].

As the HIV epidemic matures, long-term survival of HIV-infected infants to adolescence following vertical transmission is increasingly being recognised in African countries [6],[7]. Symptomatic HIV in older children and adolescents is a growing problem in clinical practice but there are few data describing the burden of HIV and its contribution to ill-health and mortality in this age group [8],[9]. In Southern Africa—the global region that has experienced the most severe adult HIV epidemic [10]—a substantial epidemic of HIV-infected long-term survivors of vertical infection is anticipated during the coming decade, with implications for adolescent health [11].

The spectrum of HIV-related disease varies considerably between adults and children, but has not been well-defined for adolescents. Although opportunistic infections predominate in all age groups, chronic noninfectious clinical conditions may be more prominent in HIV-infected adolescents than in adults and infants [9]–[12]. In clinical studies of HIV infection, children are often classified as aged 0–14 y and adults as 15–49 y, and thus distinctive features of HIV-associated morbidity among adolescents may be missed [13]–[15]. The aim of this study was to investigate the prevalence of HIV and the spectrum of morbidity among hospitalised adolescents aged 10–18 y in Harare, Zimbabwe.

## Methods

### Study Participants

Harare Central Hospital and Parirenyatwa Hospital are the main public sector hospitals in Harare and cater to two-thirds of Harare's population; the remaining one-third using private health care facilities. The cost of hospitalisation in public sector facilities is partially subsidised by the government. Malaria transmission does not occur in Harare. Between September 2007 and April 2008, patients admitted to either hospital were enrolled consecutively the following day. Recruitment was limited to weekdays and to a maximum of five patients per site per day for logistical ease. Patients aged between 10 and 18 y admitted with any acute medical or surgical condition, including trauma, were eligible. Patients were excluded if moribund (i.e., likely to die within the next few hours), requiring intensive care admission, or admitted for obstetric or elective reasons, or previously enrolled into the study.

### Data Collection

**Figure 1:**
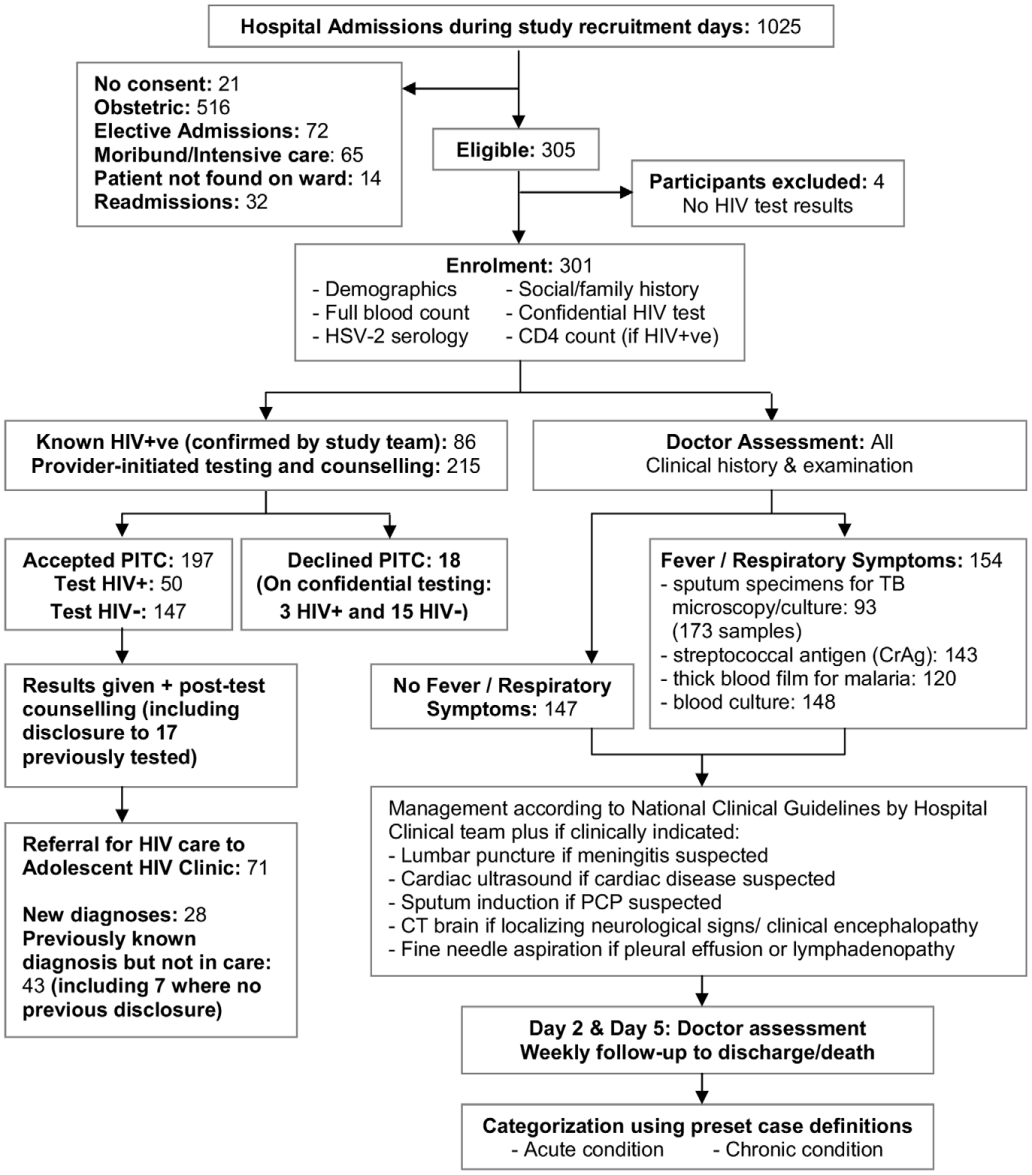
Study recruitment procedure for eligible participants.

Standardised investigations, summarised in Figure 1, included social and clinical history, height, weight, and Tanner puberty staging, and laboratory investigations (full blood count, herpes simplex virus-2 [HSV-2] serology, provider-initiated HIV testing and counselling [PITC], and CD4+ lymphocyte [CD4+] count for HIV-infected participants). Where diagnostic HIV testing was declined, participants and their guardians were asked to consent to unreported HIV-testing for study purposes. In addition, all participants with febrile, wasting, or respiratory illness had a standardised infectious screen (blood culture, blood films for malaria, cryptococcal antigen testing [serum 1∶8 dilution], two sputum specimens, and chest radiography). If *Pneumocystis jirovecii* infection was suspected, patients underwent sputum induction with nebulised hypertonic saline.

Otherwise investigations followed standard hospital guidelines including lumbar puncture for suspected meningitis and cerebral computed tomography (CT) for focal neurological signs or suspected encephalopathy. Participants were followed for the duration of their hospital stay. Pre-set diagnostic algorithms defined the definitive or presumptive cause of admission, and any underlying chronic conditions (Text S1). The Adult World Health Organization (WHO) Classification was used to stage HIV infection [16].

### Laboratory Methods

HIV serology used parallel testing with Abbott Determine and SD Bioline. Discordant samples were resolved using Vironostika. HSV-2 antibodies were detected using recombinant IgG antigen (HerpeSelect, Focus Technologies; cut-off for positivity: OD>1.1). CD4 counts were determined by flow cytometry (CyFlow counter, Partec).

Blood culture used Myco/F Lytic (MFL, Becton Dickinson) [17], inspected daily using a handheld UV Woods lamp. Bacterial pathogens were identified by Gram staining and culture on conventional media, with biochemical tests for confirmation. Concentrated decontaminated sputum specimens and positive blood cultures were examined under fluorescent microscopy (Auramine-O) and cultured for mycobacteria (Lowenstein-Jensen media). CSF and positive blood cultures were investigated for *Cryptococcus* using India ink contrast staining, culture on Sabouraud media, and cryptococcal antigen testing (IMMY, Alpha Laboratories). Induced sputum was stained with Grocott's silver stain. Thick blood films for malaria were examined using Giemsa staining.

### Statistical Analysis

Data were analysed with STATA software, version 10.0 (STATA Corporation). Continuous variables were compared using Student's *t*-test for normally distributed variables and Mann Whitney U test for variables not normally distributed. Categorical variables were compared using the Chi-squared (χ^2^) or Fisher's exact test as appropriate. Z-scores for height- and weight-for-age were calculated using British 1990 Growth Reference Curves, which provide data over the age of 10 y [18]. *z*-Scores of <−2 were considered to represent stunting and wasting, respectively [18].

Multivariate logistic regression was used for analysis of risk factors for mortality [19]. Risk factors were considered in two groups: socio-demographic (sex, age, orphanhood, difficulty raising clinic fees, tuberculosis [TB] or illness in a household member, food shortage, type of care-giver) and clinical (stage of HIV, previous TB, antiretroviral therapy [ART] status, stunting, wasting, pubertal delay, chronic disease, anaemia, poor performance scores, self-perceived poor health, broad diagnosis category). An initial model included socio-demographic factors and all factors that reached statistical significance at *p*<0.05 were included in a multivariate model. Factors that remained independently associated with death were retained. The association between each factor in the clinical group and death was assessed by adding each factor into the multivariate model that included the subset of independently significant socio-demographic factors. The multivariate model was built that included the subset of socio-demographic factors in the first multivariate model plus any clinical factors that were significant after adjusting for the socio-demographic factors. The final multivariate logistic regression model was reached by excluding single factors sequentially until all remaining factors were statistically significant.

### Ethical Considerations

Written consent was obtained from all participants and from guardians of participants aged below 16 y. Onward referral for HIV care services was made for all testing HIV-positive. Guardians were encouraged to disclose HIV status to HIV-infected participants who did not know their status. Ethical approval for the study protocol was obtained from the Ethics Committees of the London School of Hygiene and Tropical Medicine and the Joint Research Ethics Committee at the University of Zimbabwe, Medical Research Council of Zimbabwe (MRCZ), Harare and Parirenyatwa Hospital Ethics Committees and the Institutional Review Board of the Biomedical Research and Training Institute.

## Results

### Baseline Participant Characteristics and HIV Diagnosis

Of 1,025 total adolescent hospital admissions, 340 were eligible. 301 participants were recruited, with a refusal rate of 6% (Figure 1). HIV prevalence was 46%. The median age was 13 y (interquartile range [IQR]: 11–16), and 43% of participants were female, with no significant association of age or sex by HIV status (Table 1). Four (1.3%) participants tested HSV-2 positive, of whom two were HIV-positive. HIV-infected participants were less likely to be married (1% versus 9%, *p*<0.032), although the comparison was based on small numbers.

**Table 1:** Baseline demographic, clinical, and growth characteristics of adolescents admitted to hospital (*n* = 301 unless specified otherwise).

Characteristic	*n* (%) of Participants		*p*-Value
	HIV-Infected, *n* = 139	HIV-Negative, *n* = 162	
**Demographic**			
Median age y (IQR)^a^	13 (11–15)	13 (11–16)	0.14
Female	65 (47)	65 (40)	0.25
Orphan (single or double)	111 (80)	58 (36)	<0.001
Mother dead or known to be HIV-infected^b^	101 (73)	28 (17)	<0.001
Married	1(1)	8 (5)	0.032
Currently attending school	97 (70)	116 (72)	0.73
Previously tested for HIV	103 (74)	20 (12)	<0.001
HIV status already known by patient^c^	86 (62)	20 (12)	<0.001
**Clinical**			
Previous TB treatment	61 (44)	3 (2)	<0.001
Previously hospitalised more than once (*n* = 294)	39 (29)	21 (13)	<0.001
WHO HIV clinical stage 3/4	115 (83)	—	—
Receiving antiretroviral therapy	45 (32)	—	—
Self-rated general health “fair” or “poor”	120 (86)	49 (30)	<0.001
Poor performance scores (WHO scale 3/4)^d^	64 (46)	20 (12)	<0.001
Median CD4+ count, cells/µl (IQR) (*n* = 125)^e^^,^ a	151 (57–328)	—	—
**Growth and sexual development**			
Weight-for-age *z*<−2 (*n* = 266)^f^	88 (72)	29 (20)	<0.001
Height-for-age *z*<−2 (*n* = 263)^g^	63 (52)	32 (23)	<0.001
Body mass index *z*<−2 (*n* = 263)^g^	74 (73)	28 (27)	<0.001
Median mid-upper arm circumference, mm (IQR) (*n = 296*)^a^	172.5 (147–190.5)	204.5 (180–231)	<0.001
Tanner's stage 1/2 (those ≥14 y)	21 (15)	4 (2)	<0.001
Occurrence of menarche (females only)	18 (28)	34 (52)	0.005
Self-reported consensual sexual debut (*n* = 150)^h^	6 (9)	18 (22)	0.031
Self-reported forced sexual intercourse (*n* = 147)^i^	5 (8)	4 (5)	0.46

Data reported as percentage of patients and Chi-squared test used to compare HIV-infected and HIV-negative participants unless specified otherwise.

a Data reported as median, not as percentage of patients and Mann Whitney U test used for comparison of medians between HIV-infected and HIV-negative participants.

b HIV-infected participants: 14 living mothers HIV-infected; HIV-negative participants: five living mothers HIV-infected.

c 20 patients previously tested but HIV result not disclosed to patient.

d Scale 3, bedridden, <50% of the day during the last month; scale 4, bedridden, >50% of the day during the last month.

e Data missing for 14 patients: six deaths before sample taken, one unable to venesect (severe wasting), seven inadequate volumes.

f Data missing for 35 patients due to inability to stand: 20 too ill, eight fractured lower limb, four chronic disability, three pathology in lower limb (two joint infection/one soft tissue infection).

g Data missing for 38 patients: 35 as for ^b^, plus two unable to stand upright, one acute confusion.

h Data not available for151 patients (72 patients HIV-infected, 79 patients HIV-negative): 88 patients under 12 y, 63 patients too ill.

i Data missing for 154 patients: 151 as for f, three patients question not answered (two HIV-negative and one HIV-infected).

The median age at diagnosis of HIV infection was 12 y (IQR: 11–14). Of the 139 participants who were HIV-positive, 86 (62%) had tested prior to admission and knew their HIV status. Fifty participants tested positive following PITC; of these, 17 had tested HIV-positive previously but had not been told of their HIV infection. All guardians, however, agreed to disclosure to the participant with assistance of study counsellors. Only 18 (6%) participants declined PITC, of whom three were HIV-positive on unreported HIV testing and are likely to remain unaware of their HIV infection.

HIV-infected participants were significantly more likely to be orphans, previously treated for tuberculosis, previously hospitalised more than once, stunted, and pubertally immature (Tanner's stage 1/2 for participants aged 14 y or older, and girls less likely to have undergone menarche) (Table 1).

HIV-infected patients were profoundly immunosuppressed at presentation: 83% had WHO stage 3 or 4 disease and 58% had CD4+ T lymphocyte counts <200 cells/µl. Only 44 (43%) of the HIV-infected participants who had previously had an HIV test were on ART (for a median duration of 121 d).

### Causes of Hospitalisation and CD4+ Counts by Diagnostic Group

The most frequent diagnosis among HIV-infected participants was infection with tuberculosis, pneumonia, cryptococcosis, and blood stream infections being the most frequent diagnoses. Among HIV-negative participants, the commonest cause of admission was trauma, followed by acute exacerbations of chronic medical conditions, predominantly cardiac (Table 2). The median duration of stay in hospital for HIV-infected participants was 9 d (IQR 6–16 d) and for HIV-negative participants 7 d (IQR 4–18 d).

**Table 2:** Causes of admission among adolescents admitted to hospital.

Cause of Admission (Up to Four Causes Allowed)	Condition	*n* (%) of Participants		*p*-Value
		HIV-Infected, *n* = 139	HIV-Negative, *n* = 162	
**Infection**		96 (69)^a^	30 (19)^a^	<0.001^a^
**Mycobacterial disease**	Any mycobacterial disease	25 (18)	3 (2)	—
	Tuberculosis^b^	24	3	—
	*Mycobacterium avium*-intracellulare disease	1	0	—
**Bacterial infection**	Any bacterial infection	65 (47)	20 (12)	—
	Acute pneumonia	24	1	—
	Bronchitis	9	2	—
	Meningitis	9	2	—
	Soft tissue or bone/joint infection	2	5	—
	Enteritis	5	3	—
	Urinary tract infection	1	1	—
	ENT infection	0	2	—
	Sexually transmitted infection	1	1	—
	Blood stream infection^c^	12	1	—
	Organ abscess^d^	2	2	—
**Fungal Infection**	Any fungal infection	35 (25)	0 (0)	—
	Cryptococcosis	15	0	—
	Oesophageal candidiasis	21	0	—
	PCP	1	0	—
**Other infection**	Any other infection	8 (6)	10 (6)	—
	Malaria	2	5	—
	Viral	3	3	—
	Other^e^	3	2	—
**HIV wasting syndrome**		15 (11)^a^	—	—
**Traumatic injury**		4 (3)^a^	53 (33)^a^	<0.001
	Road-traffic accident	0	19	—
	Assault	1	4	—
	Accident in the home	3	30	—
	Complications of previous trauma	1	6	—
**Overdose/other psychiatric disorder**		1 (0.7)^a^	18 (11)^a^	<0.001^a^
**Acute surgical**		2 (1.4)^a^	15 (9)^a^	0.003^a^
**Acute medical noninfectious**		53 (48)^a^	52 (38)^a^	0.080^a^
	Stroke	4	0	—
	Cardiac failure	9	12	—
	Exacerbation of chronic condition other than cardiac/respiratory	1	16	—
	Malignancy	8	6	—
	Severe anaemia (Hb<7g/dl)	34	14	—
	Drug toxicity^f^	4	0	—
	Malnutrition	3	1	—
	Other^g^	4	12	—

a Data indicate the numbers and percentages of patients with ≥1 diagnosis in that category.

b HIV-infected: pulmonary, 12 (five smear-positive, one culture-positive only, six radiological); blood culture-positive, 4; TB meningitis, 2 (one with concurrent disseminated lymphadenopathy); unilateral pleural effusion, 3; arthritis confirmed on synovial biopsy, 1; miliary shadowing, 1; abdominal para-aortic lymphadenopathy confirmed on fine needle aspirate, 1; HIV-negative: pulmonary, 1 (smear-positive); blood culture-positive, 1; pericardial effusion, 1.

c HIV-infected: nontyphoidal *Salmonella spp*, 1; *Staphylococcus aureus*, 2; lactose-fermenting coliforms, 1; nonlactose-fermenting coliforms (except *Salmonella spp*), 1; Group D *Streptococcus*, 1; HIV-negative: *S. aureus*, 1.

d HIV-infected: empyema, 2; HIV-negative: intracranial abscess, 2.

e HIV-infected: scabies, 2; self-limiting fever, 1; HIV-negative: rheumatic fever, 1, self-limiting fever, 1.

f Stevens Johnsons syndrome secondary to cotrimoxazole, 2; hepatitis secondary to nevirapine, 1; lactic acidosis secondary to stavudine, 1.

g HIV-infected: anal fissures, 1; renal condition (one renal failure and one glomerulonephritis), 2; deep vein thrombosis, 1; HIV-negative: renal condition (two glomerulonephritis and three nephrotic syndrome), 5; dysfunctional uterine bleeding, 3; gastritis, 2; hepatitis, 1; pellagra, 1.

ENT, ear, nose, and throat; PCP, pneumocystis pneumonia.

The highest median CD4 counts were found in patients presenting with trauma or acute surgical causes conditions (455 and 628 cells/µl). Patients with blood stream infections, oesophageal candidiasis, and HIV-wasting syndrome had the lowest median CD4 counts (85, 77, and 34 cells/µl, respectively).

### Disease-Specific Microbiological Findings

113 (81%) and 41 (25%) of HIV-infected and HIV-negative admissions (*p*<0.001) met criteria for the standardised infectious screen (see Methods), of whom 20 (18%) and two (5%) had positive blood cultures, respectively. The most frequently identified pathogens in HIV-infected participants were nontyphoidal *Salmonella* species and *M. tuberculosis*. *Cryptococcus* spp. were identified in blood culture in three patients, in cerebrospinal fluid (CSF) culture in five patients, and seven patients had a positive cryptococcal antigen (CrAg) only.

There were 27 TB diagnoses of which 13 were pulmonary TB: six were sputum smear- positive, one was culture-positive only, and six were radiological diagnoses (smear- and culture-negative pulmonary disease with failure to respond to broad-spectrum antibiotics, but response to TB treatment at 1 mo). *M. tuberculosis* was identified in blood culture in five patients.

### Chronic Clinical Conditions

84 (28%) participants had underlying chronic medical conditions other than HIV (26% in HIV-infected versus 29% in HIV-negative, *p*<0.56). In addition, 70% of HIV-infected participants had chronic skin complaints, although rarely responsible for admission (Table 3). Admission as a result of acute exacerbation of a chronic condition accounted for 26 (19%) and 44 (27%) admissions in HIV-infected and HIV-negative participants, respectively (*p*<0.082). Chronic lung disease and cardiac disease were the most common serious HIV-related complications (Table 3). In HIV-negative participants, rheumatic heart disease was the most common chronic condition.

**Table 3:** Chronic conditions among adolescents admitted to hospital.

Chronic Condition	*n* (%) of Participants		*p*-Value
	HIV-Infected, *n* = 139	HIV-Negative, *n* = 162	
Chronic lung disease^a^	17 (12)	0 (0)	<0.001
Cardiac disease	9 (6)	12(7)	0.72
Rheumatic heart disease	0	9	—
Cor-pulmonale^b^	0	0	—
Dilated cardiomyopathy	9	1	—
Other	1	2	—
Diabetes	0 (0)	7 (4)	0.017
Epilepsy	0 (0)	3 (2)	0.093
Asthma	0 (0)	4 (2)	0.093
Chronic skin disease	97 (70)	11 (7)	<0.001
Other chronic	23 (17)	25 (15)	0.63
Neurological	7	2	—
Malignancy^c^	8	6	—
Haematological	1	3	—
Chronic infection/inflammation	1	5	—
Blindness	4	0	—
Polyarthritis	1	2	—
Congenital	1	7	—

a Static radiological appearance of focal scarring, and/or opacification, and/or cystic changes, and/or bronchial wall thickening, plus negative TB smears and cultures from current episode, plus ≥2 of the following: clubbing, recurrent (at least two) episodes of cough productive of copious amounts of purulent sputum in the last 3 mo, persistent fine basal crepitations, and/or wheezes on auscultation, cor pulmonale.

b Meets above case definition for chronic lung disease plus echocardiographic finding of right ventricular enlargement or ≥2 of the following: ascites, hepatomegaly, raised jugular venous pressure, ankle oedema.

c HIV-infected: 4, Kaposi Sarcoma; 2, Non-Hodgkins's lymphoma; 1, osteogenic sarcoma; 1, cholangiocarcinoma. HIV-negative: 3, haematological malignancy; 2, intracranial tumours; 1, myxoid neurofibroma.

### Causes of and Risk Factors for Death in Hospital

32/139 (23%) HIV-infected participants died in hospital compared with 11/162 (7%) HIV-negative participants (sex- and age-adjusted odds ratio [OR] for death for HIV-infected patients 3.7, 95% [confidence interval] CI 1.7–7.8, *p*<0.001). Death was significantly associated with low CD4+ T lymphocyte count (74 versus 183 cells/µl, *p*<0.0029). The highest case-fatality rates among HIV-infected participants were from HIV wasting syndrome (53%), any malignancy (50%), and drug toxicity (50%), and among HIV-negative participants the highest case-fatality rates were for malignancy (83%) (Table 4).

**Table 4:** Causes of death among adolescents admitted to hospital.

Cause of Death	*n* (%) of Deaths		*n* Died/Total (Case Fatality Rate)
	HIV-Infected (*n* = 32)	HIV-Negative (*n* = 11)	
Tuberculosis	4 (13)	1 (9)	5/27 (19)
Pneumonia	3 (9)	1 (9)	4/25 (16)
Meningitis	2 (6)	1 (9)	3/11 (27)
Bloodstream infection	2 (6)	0	2/13 (15)
Other infection^a^	1 (3)	1 (9)	2/11 (18)
Cryptococcosis	6 (19)	0	6/15 (40)
HIV wasting syndrome	8 (25)	0	8/15 (53)
Drug toxicity	2 (6)	0	2/4 (50)
Malignancy	4 (13)	5 (46)	9/14 (64)
Cardiac failure	0	2 (18)	2/21 (10)

The median time to death was 12 d (IQR 4–21 d) in HIV-infected and 10 d (IQR 6–20 d) in HIV-negative participants.

a HIV-infected, empyema; HIV-negative, osteomyelitis.

Of the considered socio-demographic risk factors, younger age and having a primary care-giver who was not the parent were associated with an increased risk of death (Table 5). After adjusting for these factors, advanced HIV disease, severe anaemia, TB treatment in the past, poor self-rated health, a chronic disease (except chronic skin disease), pubertal delay (Tanner puberty stage 1/2 in those aged 14 y or above), poor performance scores, wasting, and stunting were all associated with increased risk of death. In the final multivariate model, WHO stage 4 HIV infection (OR 2.8, 95% CI 1.1–7.1; *p*<0.032), chronic disease (OR 2.8, 95% CI 1.3–6.0; *p*<0.009), pubertal delay (OR 4.0, 95% C.I 1.4–11.6; *p*<0.011), and poor performance scores (OR 6.9, 95% C.I 3.0–15.8; *p*<0.001) remained independently associated with increased risk of death.

**Table 5:** Risk factors for death among adolescents admitted to hospital (*n* = 301 unless specified).

Variable	*n* Died/Total (%)	Univariate OR (95% CI)^a^	*p*-Value	Final Multivariate OR^b^ (95% CI)	*p*-Value
**Sociodemographic**					
Age					
15–18 y	5/84 (6)	1.0		1.0	
12 to <15 y	27/129 (21)	4.2 (1.5–11.4)	0.005	2.5 (0.8–7.4)	0.11
<12 y	11/88 (13)	2.3 (0.7–6.8)	0.15	2.0 (0.6–7.0)	0.25
**Primary caregiver**					
Biological parent	12/126 (10)	1.0		1.0	
Not biological parent	31/175 (18)	2.0 (1.0–4.2)	0.05	1.3 (0.5–2.9)	0.60
**Clinical**					
***HIV WHO stage***					
HIV-negative	11/162 (7)	1.0	—	1.0	—
HIV-infected and stage 1/2	2/24 (8)	1.2 (0.3–5.7)	0.85	1.0 (0.2–5.7)	0.99
HIV-infected and stage 3	5/36 (14)	1.6 (0.5–5.1)	0.42	1.6 (0.5–5.4)	0.49
HIV-infected and stage 4	25/79 (32)	5.1 (2.3–11.4)	0.0001	2.8 (1.1–7.1)	0.03
***Previously treated for TB***					
Yes	25/237 (11)	1.0	—	1.0	—
No	18/64 (28)	2.4 (1.2–4.9)	0.02	1.3 (0.6–3.2)	0.53
***Poor performance score (WHO scale 3/4)***					
No	12/217 (6)	1.0	—	1.0	—
Yes	31/84 (37)	8.2 (3.9–17.3)	0.0001	6.9 (3.0–15.8)	0.0001
***Self-perceived poor health***					
No	8/132 (6)	1.0	—	1.0	—
Yes	35/169 (21)	3.0 (1.3–6.9)	0.01	0.4 (0.1–1.5)	0.19
***Pubertal delay (Tanner's stage 1/2 in those ≥14 y)***					
No	34/276 (12)	1.0	—	1.0	—
Yes	9/25 (36)	3.1 (1.2–8.1)	0.021	4.0 (1.4–11.6)	0.01
***Stunting (height-for-age z<−2) (n = 263)***					
No	11/168 (7)	1.0	—	1.0	—
Yes	17/95 (18)	3.1 (1.3–7.3)	0.01	1.2 (0.5–3.2)	0.69
***Wasting (weight-for-age z<−2) (n = 266)***					
No	6/149 (4)	1.0	—	1.0	—
Yes	23/117 (20)	5.2 (2.0–13.7)	0.001	2.1 (0.6–6.9)	0.23
***Chronic disease (other than skin disease)***					
No	22/217 (10)	1.0	—	1.0	—
Yes	21/84 (25)	2.7 (1.3–5.3)	0.005	2.8 (1.3–6.0)	0.009
***Haemoglobin (n = 282)***					
>11g/dl	12/124 (10)	1.0	—	1.0	—
7.0–10.9 g/dl	13/110 (12)	1.0 (0.4–2.4)	0.97	0.4 (0.1–1.1)	0.07
<7.0 g/dl	14/48 (29)	3.6 (1.5–8.7)	0.005	0.8 (0.3–2.5)	0.72

a Adjusted for age group and type of primary caregiver.

b Adjusted for HIV stage, chronic disease, pubertal delay, and performance score.

## Discussion

The main finding of this study is that HIV is now the single most common cause of acute admission and in-hospital death among adolescents in Harare. HIV-infected adolescents were profoundly immunosuppressed, and the median CD4 count (51 cells/µl) was similar to that reported in other studies of hospitalised African adults in the pre-ART era [20],[21]. The spectrum of HIV-associated infections was also similar to that reported in African adults in the pre-ART era [15]. However, adolescents had an additional and heavy burden of chronic complications—such as growth failure and lung and cardiac disease—that have typically been reported in vertically HIV-infected children [22]–[24]. In this study, underlying chronic complications were associated with an increased risk of in-hospital death.

We found an equal sex distribution of HIV infection, low rates of self-reported sexual debut, and a much lower prevalence of HSV-2 infection than would be anticipated for sexually acquired HIV in southern Africans [25]. These findings, along with strong associations with orphanhood and the chronic complications discussed above, and negative association with marriage (given that marriage at young age increases risk of HIV for young African girls [26]), all favour long-term survival following mother-to-child transmission as the source of HIV infection in most of our participants. Also notable is the fact that Zimbabwe has had an unusually good safety record for preventing percutaneous HIV from early on in the HIV epidemic [27], and population-based HIV prevalence surveys among older children in southern Africa have documented substantial HIV prevalence rates with equal gender distribution [28],[29]. The widely held perception is that HIV progresses rapidly in HIV-infected infants with a median survival of 2 y [30], and thus survival to late childhood and adolescence with untreated HIV has been considered extremely unusual. Our study, however, demonstrates that long-term survival occurs and is now a major cause of morbidity among adolescents.

Diagnosis of HIV infection was frequently delayed in this age group until presentation with advanced HIV disease. A substantial minority had not been diagnosed with HIV before hospitalisation, and most others reported relatively recent diagnosis following a prolonged history of recurrent infections. The beneficial effects of early diagnosis and ART on reducing the risk of opportunistic infections and chronic complications, reducing mortality, and improving growth are well-recognised in children [31]. In addition to the high risk of irreversible complications, older age at diagnosis and delay in starting ART may potentially result in suboptimal immune response [32],[33] and blunted catch-up growth and pubertal development [34],[35].

Although the phenomenon of long-term survival in HIV-infected infants is well recognised in Zimbabwe, several factors unique to this age group may be creating both demand and supply-side barriers to diagnostic testing: in contrast to adults, there are no free-standing counselling and testing services for under 16-y-olds in Zimbabwe, and there are also legal barriers to testing without the permission of the legal guardian, who may be incapacitated or absent. Reluctance of guardians to disclose the true nature of the underlying illness was also apparent in that a substantial minority of our HIV-infected participants had not been told their previous test results. Clear advice to health professionals to offer PITC and to assist guardians with disclosure may improve timely diagnosis and adherence to subsequent ART [36].

Our study was hospital-based and therefore likely to be biased towards sicker patients and more severe HIV-associated complications. One of the study sites was based at a referral hospital, which may have resulted in a higher proportion of specialist diagnoses such as cancer. Both hospitals had ART clinics, but only nine (6%) of the HIV-infected participants were admitted from these. The vast majority of our participants (87%) were referred directly from primary health care clinics or through hospital casualty departments, and the only local alternatives to these two hospitals are private facilities. Our results are, therefore, likely to be representative of the pattern of acute severe morbidity and mortality in Harare. Judging by the high numbers of adolescents attending HIV care clinics throughout Zimbabwe [37], our results may also be more generally representative. Zimbabwe is unusual in having had high HIV prevalence in antenatal clinic attendees from the early 1990s [38], and so may be a few years ahead of other countries in the region with respect to the subsequent epidemic of adolescent survivors.

HIV-related morbidity and mortality, most likely reflecting long-term survival from the paediatric HIV epidemic in the 1990s, is now a major cause of adolescent morbidity and mortality in Harare. Recognition of the high burden of HIV in acutely unwell older children and adolescents is needed to stimulate earlier diagnosis and improve access to HIV care for this previously neglected age group. Our results strongly support implementation of PITC for all patients in this age group attending health facilities, ideally at the primary care as well as hospital level, so that diagnosis is made and treatment can be started before patients are critically ill.

## Supporting Information

Text S1 Case definitions used during the present study.
(0.04 MB DOC)

## Abbreviations

ART: antiretroviral therapy
CI: confidence interval
HSV-2: herpes simplex virus-2
IQR: interquartile range
OR: odds ratio
PITC: provider-initiated (HIV) testing and counselling
TB: tuberculosis
WHO: World Health Organization
