# Supplementary material for: Causes of Acute Hospitalization in Adolescence: Burden and Spectrum of HIV-Related Morbidity in a Country with an Early-Onset and Severe HIV Epidemic: A Prospective Survey
Source: PLoS Med. 2010 Feb 2;7(2):e1000178. doi: 10.1371/journal.pmed.1000178 (PMC2814826; doi:10.1371/journal.pmed.1000178)
Supplement: Text S1 — Case definitions used during the present study. (0.04 MB DOC) [file pmed.1000178.s001.doc]

**Case definitions used during the present Study**

*ACUTE CONDITIONS:*

*Confirmed TB*: Two or more positive results of sputum smears or cultures or one histological specimen, plus compatible illness.

*Probable smear- and culture- negative TB*: Consistent clinical and radiological disease, failure to respond to broad-spectrum antibiotics, no other cause found during investigations, and clinico-radiological response to TB treatment by one month.

*Bacterial pneumonia*: Acute onset (≤ 1week) of symptoms that included ≥2 of the following: fever, purulent sputum, leucocytosis, pleuritic chest pain plus airspace consolidation on chest radiograph performed at presentation, as well as a clinical response to broad-spectrum antibiotics.

*LRTI:* Same as definition of bacterial pneumonia, but with no radiological changes.

*Definite PCP*: *Pneumocystis* organisms detected in induced sputum specimens.

*Possible PCP:* HIV infection, exertional dyspnoea, elevated respiratory rate at rest (>25/min), bilateral mid-zone ground-glass or interstitial shadowing, radiological and clinical response to high-dose trimethoprim-sulfamethoxazole.

*Bacterial meningitis*: Conventional pathogen identified in CSF by microscopy or if CSF leucocytosis (>50cells/mm^3^) that were predominantly (>80%) neutrophils, plus clinical response to antibiotics

*Definite cryptococcosis*: *Cryptococcus*  isolated from blood or CSF samples, plus clinical response to fluconazole.

*Probable cryptococcosis*: HIV infection, positive result of serum cryptococcal antigen test at 1:8 dilution or higher plus response to fluconazole treatment.

*Septicaemia*: Isolation of clinically significant bacterial pathogen from blood culture plus clinical illness compatible with pathogen isolate.

*Enteritis*: Acute onset (≤ 1 week) of diarrhoea (3 or more loose stools /day).

*Drug Toxicity:* History of exposure to appropriate drug (≤1 week before onset of symptoms) plus recognised adverse reaction to drug (e.g AZT/anaemia ) or Stevens Johnson syndrome or new rash or anaphylaxis plus no other cause for the symptoms.

*Oesophageal candidiasis*: Oral candidiasis and recent onset of retrosternal chest pain on swallowing, plus response to treatment with fluconazole.

*HIV Wasting Syndrome*: ≥ weight loss or cachexia, with diarrhoea or fever, or both, for at least one month, not known to be due to a condition unrelated to HIV infection, plus negative investigations for TB.

*Urinary Tract Infection:* ≥2 of dysuria, fever, flank tenderness plus no abnormal vaginal or urethral discharge.

*Sexually Transmitted Infection:* Vaginal discharge and or lower abdominal tenderness (women) / urethral discharge and/or dysuria (men), or genital sore/ vesicle / ulcer.

*Kaposi Sarcoma*: Characteristic gross appearance of erythematous or violaceous plaque-like lesion on skin or mucous membrane and/or characteristic histological appearance of lesion.

*Stroke:* Sudden or rapid onset of focal neurological deficit, plus compatible CT appearance of stroke (bleed or infarct and no space occupying lesion) and no history of fevers or constitutional symptoms or chest radiographic features suggesting TB.

*CHRONIC CONDITIONS:*

*Cardiac disease:* Clinical finding of cardiac failure and evidence of cardiomyopathy, valve lesions, or pericardial disease on cardiac ultrasound.

*Chronic lung disease:* Static radiological appearance of focal scarring, and/or opacification and/or cystic changes and/or bronchial wall thickening, plus negative TB smears & cultures from current episode, plus ≥2 of the following: Clubbing, recurrent (at least 2) episodes of cough productive of copious amounts of purulent sputum, persistent fine basal crepitations and/or wheezes on auscultation, cor pulmonale.

*HIV-associated encephalopathy*: HIV infection, CT scan appearance of generalised cortical atrophy and no space occupying lesion or localised infarct, plus each of the following: (1) ≥2 of the following: hyperreflexia, palmo-mental reflex, memory loss (e.g forgets names, places, conversations), apathy, slowness of thinking (2) no other cause found to explain the clinical findings and, (3) course of illness over weeks to months.

*Diabetes*: Previous diagnosis of diabetes mellitus made by a physician or blood glucose stix >10mmol/l (for the first time) plus ≥2 of the following: Ketones in urine, ≥2+ glucose in urine, blood glucose stix >10mmol/l, shallow and fast breathing, polyuria and excessive thirst.

*Bronchial Asthma*: With or without accompanying respiratory tract infection: history of recurrent wheeze or chest tightness on waking, sleeping or during exercise; audible wheeze at examination and symptomatic relief achieved through use of salbutamol.

*Epilepsy:* Witnessed seizure or fit plus all of the following: Diagnosis of epilepsy made by a physician and history of recurrent (at least 2) seizures unprovoked by alcohol/drugs.
